# Food sensitisation patterns measured by ISAC multiplex assay in the Netherlands

**DOI:** 10.1186/2045-7022-5-S3-P126

**Published:** 2015-03-30

**Authors:** Mark Blankestijn, Rob Klemans, Henny Otten, Heike Rockmann, Carla Bruijnzeel-Koomen, Edward Knol, André Knulst

**Affiliations:** 1 University Medical Center Utrecht, Utrecht, The Netherlands

## Background

Component-resolved diagnostics is increasingly used in food allergy diagnostics. In a Dutch tertiary patient population we determined the sensitization patterns and protein families involved in our population using the multiplex Immuno-Solid phase Allergen Chip (ISAC).

## Methods

Multiplex ISAC tests were performed in case of suspicion of (extensive) poly-sensitization in patients referred for food allergy and/or atopic eczema. Patient history of 40 random patients who have had an ISAC was analysed to determine the clinical value of the ISAC.

## Results

Of 943 ISAC tests, 852 showed sensitization to at least one component and were included for further analysis. Mean age was 33,4 years. The majority of patients in our population was poly-sensitized, with 66% recognizing more than ten components. The most recognized component was Bet v 1 (70%). The top 10 food allergens were: apple (Mal d 1; 65%), peach (Pru p 1; 63%), hazelnut (Cor a 1.0401; 63%), peanut (Ara h 8; 56%, Ara h 6; 23%, Ara h 2; 15%), soy (Gly m 4; 39%), celery (Api g 1; 25%), kiwi (Act d 8; 20%) and walnut (Jug r 2; 15%). 84% showed sensitization to one or more food allergens. Within this group, 84% recognized at least one PR-10 component, followed by storage proteins (35%) and non-specific lipid transfer proteins (nsLTP; 12%). The prevalence of sensitization to cross-reactive carbohydrate determinants (CCD) within the entire population was 9%. Sensitization to at least one food components of animal origin (cow's milk/egg) was seen in 19% of all cases, with Bos d 8 recognized most frequently (8.1%). The ISAC seems to support clinical suspicion of food allergy in most cases, although sensitization together with a negative patient history is common.

## Conclusions

Bet v 1 is the allergen component recognized the most, followed closely by other PR-10 family proteins from plant foods. Sensitization to food components of animal origin was seen in 19% of the patients.

